# An Approach to Characterizing the Complicated Sequential Metabolism of Salidroside in Rats

**DOI:** 10.3390/molecules21060706

**Published:** 2016-05-30

**Authors:** Zhiqiang Luo, Xiaoyun Ma, Yang Liu, Lina Lu, Ruirui Yang, Guohua Yu, Mohan Sun, Shaokun Xin, Simin Tian, Xinjing Chen, Haiyu Zhao

**Affiliations:** 1 School of Chinese Materia Medica, Beijing University of Chinese Medicine, Beijing 100102, China; lzq4y3r@126.com (Z.L.); sijidehuaiye@126.com (X.M.); luludc399@163.com (L.L.); yangrr5021@126.com (R.Y.); sufei_sophie@163.com (G.Y.); m13001147155@163.com (M.S.); tiansimin1990@163.com (S.T.); chxj9208@sina.com (X.C.); 2 School of Traditional Chinese Medicine, Capital Medical University, Beijing 100069, China; xyyzxxx@163.com; 3 Institute of Chinese Materia Medica, China Academy of Chinese Medical Sciences, Beijing 100700, China

**Keywords:** salidroside, sequential metabolism, *in situ* closed-loop

## Abstract

Metabolic study of bioactive compounds that undergo a dynamic and sequential process of metabolism is still a great challenge. Salidroside, one of the most active ingredients of *Rhodiola crenulata*, can be metabolized in different sites before being absorbed into the systemic blood stream. This study proposed an approach for describing the sequential biotransformation process of salidroside based on comparative analysis. *In vitro* incubation, *in situ* closed-loop and *in vivo* blood sampling were used to determine the relative contribution of each site to the total metabolism of salidroside. The results showed that salidroside was stable in digestive juice, and it was metabolized primarily by the liver and the intestinal flora and to a lesser extent by the gut wall. The sequential metabolism method described in this study could be a general approach to characterizing the metabolic routes in the digestive system for natural products.

## 1. Introduction

**Figure 1:**
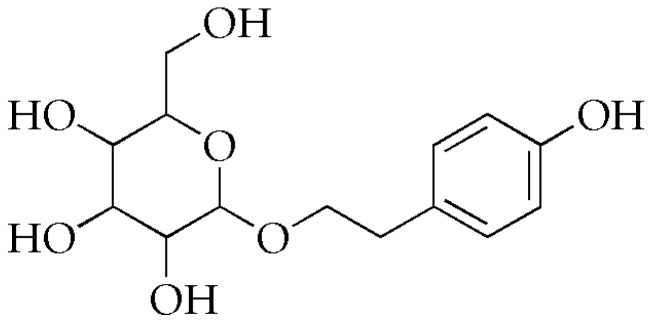
Chemical structure of salidroside.

Salidroside (*p*-hydroxyphenethyl-beta-d-glucoside), a compound with a chemical structure of phenol glycosides (Figure 1), has been identified as one of the most potent ingredients isolated from *Rhodiola crenulata* [1]. Salidroside reportedly possesses broad pharmacological activities, such as resisting anoxia [2], anti-fatigue [3], anti-inflammation [4,5], anti-aging [6], anti-cancer [7,8,9], antioxidation [10], cardioprotective [11], and hepatoprotective [12] properties. As a well-known natural product, salidroside is widely used as a bioactive ingredient for functional foods, dietary supplements, and phytomedicines around the world [13,14,15,16].

Although salidroside exhibited a wide variety of biological activities in *in vitro* and *in vivo* experiments, only a few reports regarding the metabolism and disposition of salidroside are available. Han *et al.* developed a LC/MS method to identify salidroside and its major metabolites in rat plasma, bile, urine and feces, and up to 7 metabolites were detected. Among them, sulfate and glucuronidation conjugation were the primary pathway for salidroside [17]. Hu *et al.* identified eight metabolites of salidroside from the rat urine samples via ultra-performance liquid chromatography coupled with quadrupole time-of-flight mass spectrometry and high performance liquid chromatography coupled with quadrupole-linear ion trap mass spectrometry [18]. Guo *et al.* studied the metabolism of salidroside to its aglycone *p*-Tyrosol in rats [19]. However, the existing results could not provide a comprehensive map of the dynamic biotransformation process of salidroside following oral administration. Accordingly, it is necessary to study the sequential metabolic changes of salidroside *in vivo.*

Hence, the primary goal of this study was to develop a holistic, sequential and reproducible approach to describe the complicated sequential metabolism of salidroside before absorption into the systemic blood stream. In brief, the approach includes four fundamental steps: (1) enzymatic transformation in digestive juice, (2) biotransformation by intestinal flora tract, (3) gut wall metabolism, and (4) hepatic metabolism [20]. The approach was based on comparative analysis and a carefully designed mix of metabolic techniques such as *in vitro* incubation, *in situ* closed-loop and *in vivo* blood sampling. Ultra-high-pressure liquid chromatography coupled with linear ion trap-Orbitrap tandem mass spectrometry (UHPLC-LTQ-Orbitrap) was used to analyze the metabolites of salidroside in different metabolic sites. High-resolution mass spectrometry such as LTQ-Orbitrap plays a crucial role in metabolic analyses owing to its accurate MS data and reliable metabolite identification when attempting to discriminate metabolites with different MS/MS fragments [21]. By the results, we can determine the probable metabolic pathways of salidroside and the sequential contribution of each metabolic site. Research on the sequential metabolism of salidroside would provide helpful information on how the biological activities of these components are changed before these components are delivered to the target sites.

## 2. Results and Discussion

### 2.1. Stability of Salidroside in Artificial Gastric Juice and Intestinal Juice

Salidroside was quite stable in artificial gastric juice and intestinal juice. The content of salidroside was not altered significantly according to the peak areas during *in vitro* incubation with digestive juices (RSD < 5.0%).

### 2.2. Fragmentation of Salidroside Standard

The chromatographic and mass spectrometric conditions were optimized using salidroside standard. Metabolite characterization was on the basis of the fragmentation patterns of salidroside.

**Figure 2:**
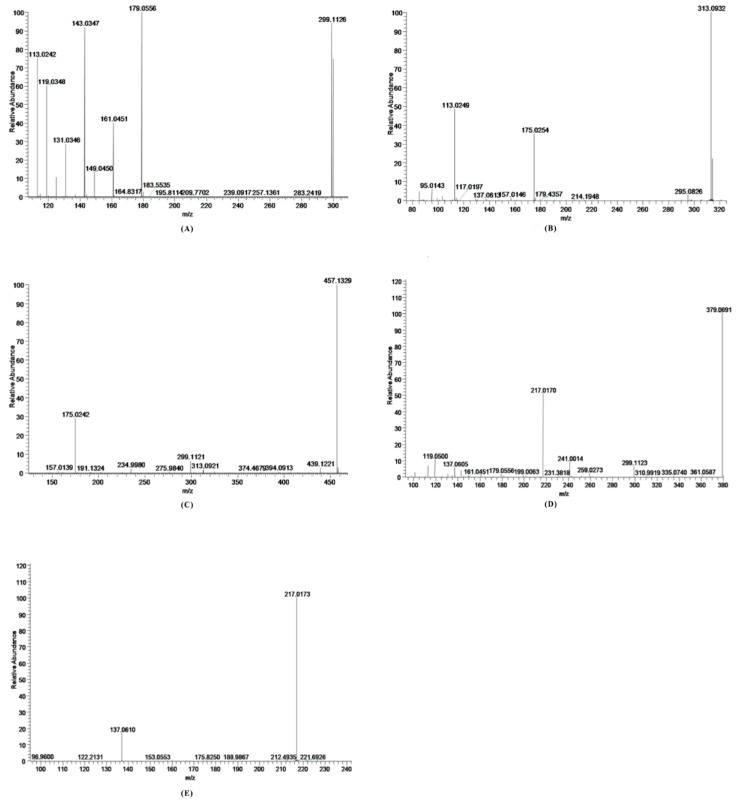
Representative MS/MS spectrum: (**A**) M0 (*m*/*z* 299); (**B**) M1 (*m*/*z* 313); (**C**) M2 (*m*/*z* 475); (**D**) M3 (*m*/*z* 379); (**E**) M4 (*m*/*z* 217).

As shown in Figure 2A, the deprotonated molecular ion of salidroside at *m*/*z* 299.1126 gave rise to six major product ions at *m*/*z* 113.0242, 119.0348, 131.0346, 143.0347, 161.0451, 179.0556. Two important fragmentations from deprotonated salidroside were the cleavage of the glycosidic linkage leading to the formation of *m*/*z* 179.0556 and 119.0348. The fragment at *m*/*z* 161.0451 was generated through the loss of H_2_O from the ion at *m*/*z* 179.0556, and then produced the ion at *m*/*z* 143.0347 by the further elimination of H_2_O. The fragment ion at *m*/*z* 131.0346 was rationalized to originate from the product ion at *m*/*z* 179.0556 through the loss of one molecule of CH_2_O and one molecule of H_2_O. Subsequently, the predominant fragmentation ion at *m*/*z* 113.0242 was produced by the reduction of H_2_O from *m*/*z* 131.0346. Thus, it can be concluded that the ions at *m*/*z* 113.0242, 119.0347, 131.0346, 143.0347, 161.0451, 179.0556 were the characteristic product ions of salidroside.

### 2.3. Identification of Salidroside Metabolites in Different Plasma Samples

Metabolites of salidroside were detected and identified by comparing plasma samples from salidroside-treated rats with corresponding control samples. The structures of the metabolites were elucidated by comparing their molecular weights and product ions with those of the parent drug [22]. A total of 4 metabolites were found from different plasma samples. Detailed information on salidroside (M0) and its metabolites (M1–M4) was summarized in Table 1.

#### 2.3.1. Parent Compound M0

Salidroside (M0) was identified according to the UHPLC retention time (11.17 min), the accurate MS at *m*/*z* 299.1126 [M − H]^−^, and MS/MS spectra from the authentic standard. Characteristic fragment ions at *m*/*z* 113, 119, 131, 143, 161, 179 could also be found in the MS/MS spectra.

#### 2.3.2. Metabolite M1

M1 (retention time t_R_ = 8.32 min) showed the molecular ion at *m*/*z* 313.0932 (4.5 ppm, elemental composition of C_14_H_17_O_8_), which was 176 Da higher than the *m*/*z* of the aglycone *p*-tyrosol, suggesting a glucuronic acid group was conjugated to the aglycone *p*-tyrosol [18]. According to the MS/MS spectrum (Figure 2B), two major product ions of M1 were found at *m*/*z* 175.0254 (the [M − H]^−^ ion of glucuronic acid) and 113.0249 (C_5_H_5_O_3_). The fragment at *m*/*z* 295.0826 was generated through the loss of H_2_O from the quasi-molecular ion.

#### 2.3.3. Metabolite M2

M2 (retention time t_R_ = 8.93 min) displayed an [M − H]^−^ ion at *m*/*z* 475.1464 (3.8 ppm, elemental composition of C_20_H_27_O_13_), which was 176 Da higher than the *m*/*z* of M0, indicating that it was the glucuronide conjugated product of salidroside [18]. The major fragment ion (Figure 2C) at *m*/*z* 457.1329 was a H_2_O loss from the quasi-molecular ion. The product ions at *m*/*z* 175.0242 and 299.1121 were ions of glucuronic acid and salidroside produced by M2.

#### 2.3.4. Metabolite M3

M3 (retention time t_R_ = 10.80 min) exhibited a [M − H]^−^ ion at *m*/*z* 379.0691 (−0.6 ppm, elemental composition of C_14_H_19_O_10_S), which was 80 Da heavier than that of salidroside, indicating sulfation of salidroside [18]. The major fragment ion determined at *m*/*z* 217.0170 was shaped by the elimination of Glu (162 Da). Characteristic product ions at *m*/*z* 299, 137, 119 were also found in the MS^2^ fragmentation (Figure 2D).

#### 2.3.5. Metabolite M4

M4 (retention time t_R_ = 11.61 min) exhibited a [M − H]^−^ ion at *m*/*z* 217.0173 (3.4 ppm, elemental composition of C_8_H_9_O_5_S), which was 80 Da heavier than that of the aglycone *p*-tyrosol, indicating a sulfuric acid group was M4 [18]. The major fragment ion (Figure 2E) at *m*/*z* 137.0610 was the aglycone *p*-tyrosol.

### 2.4. The Sequential Process of Salidroside in the Digestive System

**Figure 3:**
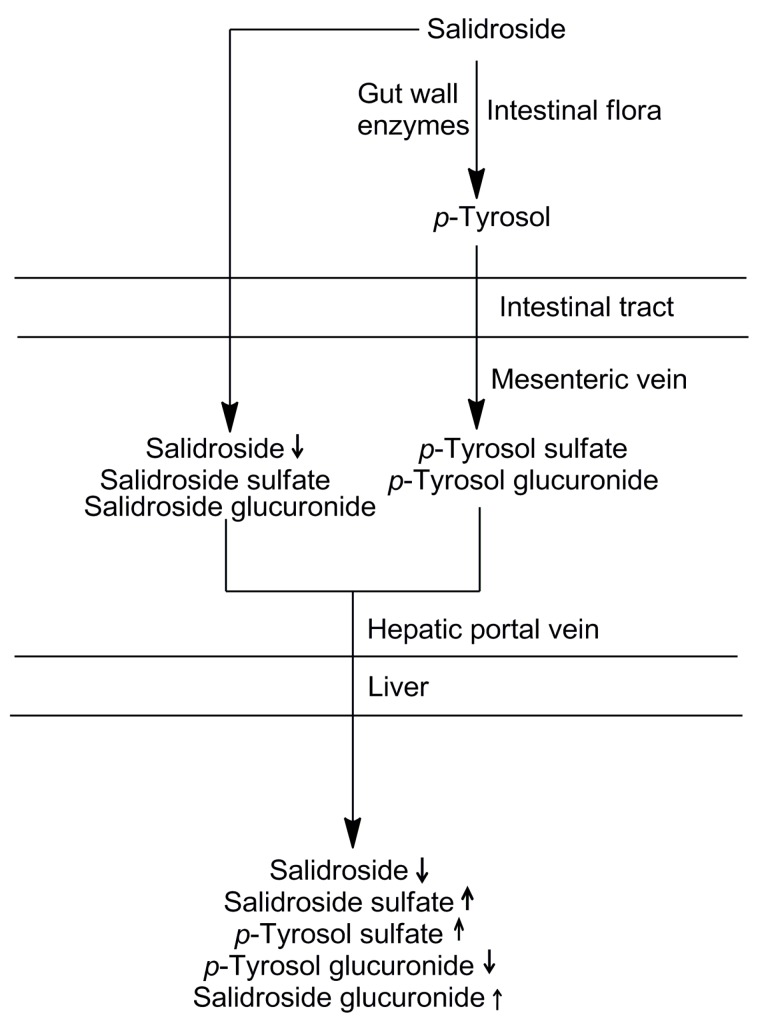
The sequential process of salidroside in the digestive system.

Using the sequential metabolism method, a total of 4 metabolites of salidroside have been detected and identified in different plasma samples. Our results indicated that glucuronidation, sulfation, and deglycosylation were the major metabolic pathways of salidroside. The contribution of each metabolic site to the overall metabolism was also shown in Table 1. When salidroside was administered orally, enzymes in the gut wall may play an important role in the initial metabolism. However, results showed that the biotransformation rate of salidroside by the gut wall was very low. It can be noted that intestinal flora tract and liver were the major metabolic sites for salidroside. Salidroside appears to undergo sequential and multiple pathways of degradation and metabolism by intestinal flora tract and liver to produce active metabolites or intermediates, thereby exerting *in vivo* pharmacological functions. The sequential process of salidroside in the digestive system is shown in Figure 3.

## 3. Experimental Section

### 3.1. Chemicals and Reagents

Salidroside (purity > 98%) was purchased from Nanjing Zelang Biological Technology Co., Ltd. (Jiangsu, China). HPLC-grade acetonitrile and formic acid were obtained from Fisher Scientific. High purity water was prepared by using the Millipore Milli Q plus purification system. All other solvents and chemicals used were of analytical grade and commercially available.

The perfusion solution was Krebs–Ringer (K–R) buffer solution, which contained 7.8 g NaCl, 0.35 g KCl, 1.37 g NaHCO_3_, 0.02 g MgCl_2_, 0.32 g NaH_2_PO_4_, and 1.48 g glucose in 1000 mL distilled water.

### 3.2. Preparation Perfusion Solution of Salidroside

The perfusion solution of 10 mg/mL salidroside was prepared by dissolving 500 mg salidroside in 50 mL K–R buffer solution.

### 3.3. Animals

Sprague-Dawley rats (male, 200–250 g) were obtained from Spfanimals Laboratory Animal Technology Co. Ltd (Beijing, China). All animal experiments were conducted using protocols approved by the University Committee on Ethics in the Care and Use of Laboratory Animals (SYXKJING(2001-0024)), and the animals were housed and handled according to the Laboratory Animal Medicine Guidelines of Beijing University of Chinese Medicine. Before the study, the rats were kept in a temperature- and humidity-controlled room (23 °C, 60% air humidity) with unlimited access to standard diet and water. The rats were acclimated for at least 7 days, then fasted overnight but supplied with water *ad libitum* prior to the date of the experiment.

### 3.4. Stability in Digestive Juice with Enzymes

Salidroside (1 mg) was added into 15 mL of artificial gastric juice (0.08 M HCl containing 50 mg of pepsin, pH 1.5) and incubated in a 37 °C shaking water bath for 0.5, 2, or 4 h, respectively. The reactions were rapidly terminated by water-saturated *n*-butanol. The mixture was centrifuged at 5000× *g* for 15 min, and then the supernatant was evaporated to dryness under a stream of nitrogen. The residue was dissolved in 500 μL of methanol, and then filtered through a 0.45 μm membrane for HPLC analysis.

Salidroside (1 mg) was added into 15 mL of artificial intestinal juice (0.05 M KH_2_PO_4_ containing 50 mg of pancreatin, pH 6.8) and incubated in a 37 °C shaking water bath for 1, 3, or 6 h, respectively. The samples were prepared as the procedure described in artificial gastric juice.

### 3.5. Intestinal Flora Metabolism Method

The *in situ* closed-loop method was extensively used to study intestinal absorption. The approach enables intestinal absorption to occur at body temperature for an appointed time. The model also allows absorption to be measured separately at different regions of rat intestine, jejunum, ileum and colon [23].

The complex surgical procedure and the *in situ* closed-loop experiments were conducted in accordance with the methods described previously [20,23]. The rats were divided into two groups. In one group, blood was collected from the mesenteric vein and the drug was injected into the gut loop directly. In the other group, blood was also collected from the mesenteric vein, but the drug was injected only after the intestinal flora had been flushed out.

Before perfusion surgical operation, five to seven rats were selected for donor blood per experiment and a total of 50–70 mL blood was obtained from the abdominal aorta with a heparinized syringe. The blood incubated in a 37 °C water bath was prepared to be transfused into the recipient rat through the jugular vein to balance the blood loss via the mesenteric vein. The recipient rat was anesthetized with chloral hydrate (400 mg/kg) and placed under a heat lamp to maintain normal body temperature. To sustain the anesthetic condition, one third of the initial dose of chloral hydrate was administered in the following experiment.

Upon verification of the loss of pain reflex, the left external jugular vein was exposed and cannulated with a 24 GBD Intima II catheter (Becton Dickinson Medical Devices Co. Ltd, Beijing, China) to transfuse blood collected previously from donor rats. Then the abdomen was opened with a midline incision and a 5 cm colon segment was isolated by two ligatures to form a loop. To collect venous outflow, a 24 G catheter with heparinized saline was inserted into the mesenteric vein draining the segment. The blood flow amounted to 0.3 mL/min to avoid blood loss.

For the study without intestinal flora, the intestinal content was washed out by perfusing the loop with prewarmed K–R buffer solution. When the flora was carefully expelled, air was pumped through the syringe to clean the lumen. Salidroside solution (2 mL) was injected into a loop with a syringe. At the conclusion of surgery, the loop of gut with blood supply intact was covered with Parafilm to reduce evaporation.

For the study with intestinal flora, the operation was the same as described above for the study without intestinal flora except for the flush process.

Mesenteric blood was collected in heparinized centrifuge tubes within 1.5 h. The blood samples were centrifuged at 5000 rpm for 15 min to obtain the plasma, and then treated with 3 volumes of methanol to precipitate protein. The mixture was vortexed for 10 min, and centrifuged at 10,000 rpm for 15 min. The organic layer was transferred to another tube, and evaporated to dryness under a stream of nitrogen at 40 °C. Then, the dried residue was dissolved in 200 μL of methanol for LC/MS analysis.

### 3.6. Gut Wall Metabolism Method

About 10 cm of jejunum was selected and the surgical approach for metabolism without intestinal flora was used. Blood samples were collected and prepared as described above.

### 3.7. Hepatic Metabolism Method

The surgical approach was the same as described above but without blood supplementation and collection. Intestinal flora metabolism and gut wall metabolism would all occur before the hepatic metabolism, and thus 5 cm colon and 10 cm jejunum were used in this experiment. Blood samples were collected from the abdominal aorta after 1.5 h and prepared as described above.

### 3.8. HPLC Analysis

The HPLC system used to analyze the stability of salidroside in digestive juice was composed of a Waters 600 controller and pump and a Waters 2487 detector measuring absorbance at 214 nm (Waters Corporation, Milford, MA, USA). The chromatographic separation of salidroside was carried out on a 250 mm × 4.6 mm C_18_ column with acetonitrile/water 80:20 (*v*/*v*) as isocratic mobile phase at 1.0 mL/min. The column temperature was set at 35 °C and the volume injected amounted to 20 μL.

### 3.9. LC/MS Analysis

Sample analyses were performed using an ultimate 3000 LC system coupled to an LTQ Orbitrapmass spectrometer via an ESI interface. The chromatography system consisted of an autosampler, a diode array detector, a column compartment and two pumps. Xcalibur, Metworks and Mass Frontier 7.0 software packages (Thermo Corporation, Waltham, MA, USA) were employed for data collection and data analysis.

Chromatographic separations were performed on a Thermo Scientific BOS Hypersil C_18_ column 2.1 × 150 mm^2^, with 2.4 μm particle size. The mobile phases A and B comprised 0.1% formic acid in water and acetonitrile, respectively. The LC gradient program (time (in min)/%mobile phase B) was set as 0.01/1, 3/1, 6/5, 12/10, 19/50, 24/85, 26/1, 30/1. The chromatographic runs were performed at a flow rate of 0.3 mL/min. The injection volume was 3 μL with column oven at 35 °C.

The ESI source parameters were as follows: the capillary temperature was 250 °C, source voltage and ispray voltage were set at 5 kV, shealth gas (N_2_) flow was 35 psi. The ESI source was operated in the negative ionization mode. In the Fourier transform (FT) cell, full MS scans were acquired in the range of *m*/*z* 50–1000. The MS/MS experiments were set as data-dependent scans.

## 4. Conclusions

In this paper, a practical approach was established to study the sequential metabolism of salidroside after oral administration. Four metabolites were found in this sequential process and their structures were elucidated according to the retention times, accurate molecular mass, and characteristic fragment ions. The metabolic pathways were proposed and the relative contribution of each metabolic site to the total metabolism of the parent was also determined. Based on the result, the sequential process of salidroside after oral administration was clearly characterized. It was found that most salidroside was converted to the glucuronide and sulfate forms of both the parent compound and the aglycone, and then transported to the target tissues through the blood stream. The results obtained in this study could provide useful information about salidroside bioactivity and beneficial health effects. Furthermore, the proposed sequential metabolism methodology could be applicable to mechanistic studies of other compounds involving complicated sequential metabolic reactions.

## Figures and Tables

**Table 1:** UHPLC-LTQ-Orbitrap analysis and relative percentage area of salidroside and four metabolites in different blood samples.

Metabolite	t_R_/min	Found (Da)	Calculated (Da)	Error (ppm)	Fragment ion *m*/*z* (Da)	Formula	Metabolite Description	Relative Percentage Area in Different Blood Samples ^1^			
								GWM ^2^	WOIF	WIIF	HM
M0	11.17	299.1126	299.1125	0.4	179.0556	C_14_H_20_O_7_	Parent	94.26%	99.59%	43.85%	22.93%
					119.0348						
					161.0451						
					143.0347						
					131.0346						
					113.0242						
M1	8.32	313.0932	313.0918	4.5	175.0254	C_14_H_18_O_8_	Deglycosylation + Glucuronidation	2.17%	0.38%	15.67%	7.43%
					113.0249						
					295.0826						
M2	8.93	475.1464	475.1446	3.8	457.1329	C_20_H_28_O_13_	Glucuronidation	0.66%	0.04%	3.52%	16.42%
					175.0242						
					299.1121						
M3	10.80	379.0691	379.0693	−0.6	217.0170	C_14_H_20_O_10_S	Sulfation	2.91%	ND	7.00%	11.00%
					299.1123						
					137.0605						
					119.0500						
M4	11.61	217.0173	217.0165	3.4	137.0610	C_8_H_9_O_5_S	Deglycosylation + Sulfation	ND ^3^	ND	29.96%	42.23%

^1^ The relative percentage area was calculated as (peak area of each metabolite/total peak area of metabolites) × 100%; ^2^ GWM, gut wall metabolism; WOIF, without intestinal flora group; WIIF, within intestinal flora group; HM, hepatic metabolism; ^3^ Not detected.
